# Use of Brain Biomechanical Models for Monitoring Impact Exposure in Contact Sports

**DOI:** 10.1007/s10439-022-02999-w

**Published:** 2022-07-22

**Authors:** Songbai Ji, Mazdak Ghajari, Haojie Mao, Reuben H. Kraft, Marzieh Hajiaghamemar, Matthew B. Panzer, Remy Willinger, Michael D. Gilchrist, Svein Kleiven, Joel D. Stitzel

**Affiliations:** 1 Department of Biomedical Engineering, Worcester Polytechnic Institute, Worcester, MA USA; 2 Dyson School of Design Engineering, Imperial College London, London, UK; 3 Department of Mechanical and Materials Engineering, Faculty of Engineering, Western University, London, ON N6A 5B9 Canada; 4 Department of Mechanical and Nuclear Engineering, Department of Biomedical Engineering, The Pennsylvania State University, University Park, PA USA; 5 Department of Biomedical Engineering, The University of Texas at San Antonio, San Antonio, TX USA; 6 Department of Mechanical and Aerospace Engineering, University of Virginia, Charlottesville, VA USA; 7 University of Strasbourg, IMFS-CNRS, 2 rue Boussingault, 67000 Strasbourg, France; 8 School of Mechanical & Materials Engineering, University College Dublin, Belfield, Dublin 4, Ireland; 9 Division of Neuronic Engineering, KTH Royal Institute of Technology, Hälsovägen 11C, 141 57 Huddinge, Sweden; 10 Department of Biomedical Engineering, Wake Forest School of Medicine, Winston-Salem, NC USA

**Keywords:** Brain biomechanics, Concussion, Subconcussion, Impact kinematics, Instrumentation, Finite element model

## Abstract

Head acceleration measurement sensors are now widely deployed in the field to monitor head kinematic exposure in contact sports. The wealth of impact kinematics data provides valuable, yet challenging, opportunities to study the biomechanical basis of mild traumatic brain injury (mTBI) and subconcussive kinematic exposure. Head impact kinematics are translated into brain mechanical responses through physics-based computational simulations using validated brain models to study the mechanisms of injury. First, this article reviews representative legacy and contemporary brain biomechanical models primarily used for blunt impact simulation. Then, it summarizes perspectives regarding the development and validation of these models, and discusses how simulation results can be interpreted to facilitate injury risk assessment and head acceleration exposure monitoring in the context of contact sports. Recommendations and consensus statements are presented on the use of validated brain models in conjunction with kinematic sensor data to understand the biomechanics of mTBI and subconcussion. Mainly, there is general consensus that validated brain models have strong potential to improve injury prediction and interpretation of subconcussive kinematic exposure over global head kinematics alone. Nevertheless, a major roadblock to this capability is the lack of sufficient data encompassing different sports, sex, age and other factors. The authors recommend further integration of sensor data and simulations with modern data science techniques to generate large datasets of exposures and predicted brain responses along with associated clinical findings. These efforts are anticipated to help better understand the biomechanical basis of mTBI and improve the effectiveness in monitoring kinematic exposure in contact sports for risk and injury mitigation purposes.

## Summary Statements

This work was part of the Consensus Head Acceleration Measurement Practices (CHAMP) project. The objective of CHAMP was to develop consensus best practices for the gathering, reporting, and analysis of head acceleration measurement data in sport. Subject matter experts were recruited to draft a series of papers on various aspects of the issue. As described in detail in a companion paper (Arbogast *et al*. 2022),^9^ each team drafted a paper and a several summary statements ahead of the CHAMP Consensus Conference, held on March 24–25, 2022 at the Children’s Hospital of Philadelphia. The following summary statements regarding brain computational modeling were discussed, revised as necessary, and ultimately approved by more than 80% of the vote at the conference:

1. Brain biomechanical models have strong potential to improve injury prediction and interpret kinematic exposure over global head kinematics alone. They provide the ability to interrogate physics-based tissue level response, estimate risk of injury, and offer insight into injury specifics such as location and extent of structural damage.
2. The modeling community advocates for the sensor community to standardize reporting of head kinematic data. Standardized reporting should include sensor hardware and software details, as well as specifics on coordinate system, post-processing, sampling frequency, and subjects’ morphological and demographic information.
3. It is recommended that model quality be assessed comprehensively by comparing with experimental data related to the metric used for model predictions (e.g. deformation, strain, stress), correlating against real-world data, and then where possible, comparing to responses from existing models. It is also recommended that models be reevaluated for validation quality when new experimental data or analytic strategies become available.
4. We recommend modelers explore modern data science techniques to efficiently process large amounts of sensor data.
5. The modeling community advocates for a curated open-access database repository to facilitate sharing of real-world data such as subject-specific head kinematics, injury diagnoses, and other associated information including head/brain morphology. In addition, simulation results from existing models using idealized kinematic profiles should be shared as a benchmark for cross-model examination.

## Introduction

This paper intends to recommend best practices related to using physics-based, computational finite element (FE) models of the human brain for predicting intracranial biomechanical responses and subsequent interpretation of sustained head impact kinematics. Topics of model development, validation, simulation, result interpretation, and limitations are discussed. Finally, a list of recommendations and consensus-derived best practices are presented. Wherever possible, all of these topics are taken in the context of interpreting intracranial biomechanical responses from instrumentation-based measurements of head impact kinematics in contact sports.

## Model Development

### Model Development Overview

**Figure 1:**
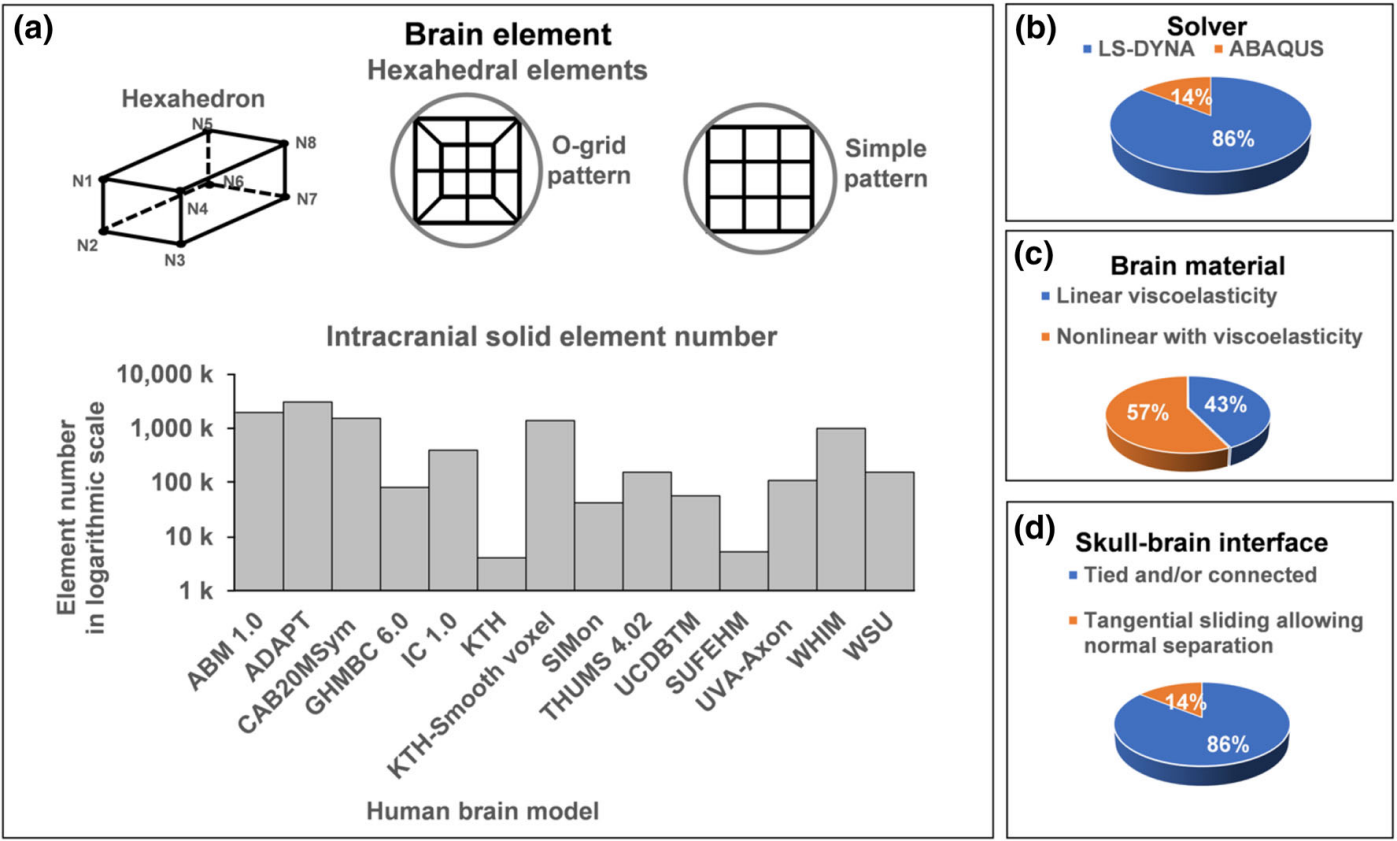
A brief overview of a number of selected brain models (see text for model acronyms). (a) All hexahedral meshes with various element numbers of the models (in logarithmic scale; WHIM shows the maximum number available^170^), (b) solvers used by the various models, (c) material model with more using nonlinear with viscoelasticity, and (d) brain-skull interface with the majority adopting tied or nodal sharing conditions.

The purpose of developing a human brain biomechanical model (hereafter ‘brain model’) is to accurately represent brain biomechanics *in silico*. This allows estimating brain responses in real-world kinematic events through computer simulations that are otherwise difficult or impossible to measure directly. Most brain models are developed using physics-based finite element (FE) method, which is a modern engineering tool widely used across industry and academia. The solution of the mechanical response of a complex FE model is resolved within a discretized mesh consisting of a collection of simple-shaped elements. Here, several critical aspects of FE model development are addressed, including mesh development, assignment of material properties, and defining boundary conditions. Several high-quality brain models are now available for estimating brain responses during blunt head kinematics. Some comparisons of these models include model validations,^32,82^ evaluation of predicted brain strains,^42^ *etc*. Figure 1 summarizes some of these models.^42^

Generating high-quality meshes of the brain is usually the first hurdle in model development. This is critical for representing the complex brain anatomy and providing accurate element-level response predictions. In the literature, 8-noded hexahedral meshes are usually preferred to represent the brain,^63^ which often incorporate an o-grid or “butterfly” pattern to represent rounded surfaces (Fig. 1a). Together with selectively reduced integration and hourglass control, linear hexahedral elements provide sufficient accuracy and efficiency using explicit time integration in LS-Dyna (Ansys, Canonsburg, PA)^54,62,80,99,114,122,135,144,155,166^ or ABAQUS (Dassault Systèmes, France)^149,172^ solvers (Fig. 1b). Higher order tetrahedral elements are also possible for meshing the brain,^117^ but they can suffer in efficiency.^63^ Brain models with various numbers of elements ranging from five thousand to three million have been reported (Fig. 1a). The effects of element number on predicting brain strains have also been quantified.^63,170^ Thin shell elements have been used to represent the falx and tentorium that are important to support the brain in rotational motion. Other components include ventricles that are sometimes approximated as fluid elements or low-shear solids and skull-to-brain membranes that are usually overlaid to nearby solid elements.

After meshing, material properties are assigned to each element to represent brain anatomical structures. Constitutive model assumptions and parameters are often periodically updated as new experimental data become available. Both linear viscoelastic^80,99,114,122,144,166^ and nonlinear, visco-hyperelastic^54,62,99,135,144,149,155,172^ material properties have been commonly used (Fig. 1c). Typically, the brain is assumed to have a density close to water, and is nearly incompressible with the bulk modulus many orders of magnitude greater than the shear modulus (GPa vs. kPa). This combination of material characteristics is important for accurate prediction of intracranial pressure gradients during translational head motion. In addition to the brain parenchyma, membrane structures including the falx, tentorium, dura, arachnoid, and pia if included are usually defined as elastic materials. Due to the rather large variation in material property characterization of the brain,^23,168^ it is common for model developers to optimize material parameters to improve model validation while balancing numerical stability and efficiency for practical use.

Defining appropriate boundary and interface conditions between the brain and skull, and among internal brain structures remains a challenge because these areas lack abundant experimental data to support modeling choices. A common practice is to use tied conditions or nodal sharing,^54,62,80,114,122,135,144,155,166,172^ as used in the majority of models (Fig. 1d). A tangential-sliding-only interface condition is also possible, allowing sliding motion between the skull and brain.^99,149^ Similar to brain material property assignment, it is also common to select and optimize brain-skull boundary conditions to satisfy model validation. Nevertheless, there remains concern that a tied brain–skull boundary condition is not a perfect anatomical representation,^183^ even if it may produce reasonable model validation against cadaveric data deep to the brain.^72^ There has been limited investigation on the relative motion between the brain-skull interface and the resulting strain at the outer surface of brain models.^122^

It is noteworthy that new capabilities are continually adopted to improve a model’s sophistication and predictive power. The stiffening effect of blood vessels, which are small in volume percentage but have a stiffness value orders of magnitude higher than the brain, has been studied by incorporating the cerebral vasculature network.^76,165,173,174^ White matter fiber tracts have also been modeled either through material definition^59,136,171^ or explicitly embedded in the brain to study axon loadings.^102,155,178^ Fluid–solid interaction between brain and cerebrospinal fluid (CSF) has also been investigated,^38,181^ which is an alternative to assigning low shear modulus fluids to the CSF. Lastly, brain models with detailed meshes with millions of elements are also available, offering an improved look at brain anatomy such as gyri and sulci or direct correlation with neuroimaging.^54,62,105,122^

### Selected Models and Applications

This section reviews some legacy brain models and more contemporary ones at the global organ level, their unique features and applications, ordered by the year each developer group first published their initial versions. This list is not inclusive of every brain model ever developed and was compiled by the authors. It aims to avoid endorsement of any model over any other.

The Wayne State University Head model (WSUHM) was developed based on earlier versions in 1993^134^ and 1995.^179^ It was one of the first models used to study concussions in American football.^167^ Pressure and shear stress were predicted across the brain in 12 National Football League (NFL) impacts involving 24 players, with 9 players diagnosed with concussion. The study found intracranial pressure to be more strongly correlated with translational acceleration than rotational acceleration. In addition, large shear stress in the brainstem and thalamus was identified. These are areas that are often damaged in traumatic brain injury (TBI), particularly in conjunction with diffuse axonal injury (DAI), and are also relevant in concussion. The study showed shear stress in the upper brainstem can better predict concussion than pressure, translational acceleration, rotational acceleration and Head Injury Criterion (HIC).^150^ Later, the model was used to predict strain distribution in the brain in 28 NFL collisions (22 concussions).^151^ It was shown that midbrain strain and strain rate correlated with memory and cognitive deficits and removal from play.

The Strasbourg University FE Head Model (SUFEHM) has been used to determine injury metrics for predicting the risk of concussion, subdural hematoma (SDH) and skull fractures. It originated from work in 1997.^88^ This visco-elastic brain model has been applied to the replication of 28 NFL collisions^153^ and 125 real world head trauma^31^ to derive brain tolerance limits. A SUFEHM-Box pipeline has been made available for end users for assessment of head protection systems in the automotive,^56,126^ motorcycle,^17,145^ and bike and equestrian helmet industries.^15,30,71^ SUFEHM is part of “Certimoov.com” helmet rating and used for front car crash safety assessment within EuroNCAP. A deep learning version of the model was developed for helmet applications.^16^ An anisotropic version with visco-hyperelastic material for white matter from diffusion tensor imaging (DTI) data has been developed.^24,25^ This was validated^135^ and used to derive prediction for skull fracture^137^ and mild TBI.^138^ The prediction capability of multiple metrics including maximum axonal strain (MAS), strain rate, cumulative strain damage metric (CSDM), *etc*. showed axonal strain prediction capability.

Several versions of the KTH (The Kungliga Tekniska Högskolan Royal Institute of Technology) models exist. An early version with visco-hyperelastic brain material properties was used to simulate 58 NFL collisions and to determine the mechanical parameter for better concussion prediction.^98^ Later, Giordano and Kleiven^59^ incorporated anisotropic material properties for white matter to test the prediction accuracy of MPS, MAS, CSDM, HIC and BrIC. They found that they are all significant predictors of concussion based on logistic regression. The model was also used for multiscale modeling of TBI^26^ and to incorporate white matter mechanical anisotropy and assess its effects on the predictions.^57,58^ Another version incorporating several pairs of bridging veins has shown large strains in these veins under head rotations, suggesting that large rotational accelerations can produce SDH.^180,182^

A smoothed voxel KTH version was also developed from MRI,^75,77,78^ which was validated by segmentation accuracy, element quality and brain motion. It showed that maximal strain was concentrated in the depth of sulci during rotational loading, similar to reported locations of DAI.^77^ Therefore, it was suggested that the inclusion of sulci may be considered for future FE head models.

The ADAPT model^105,124^ (A Detailed and Personalizable Head Model with Axons for Injury Prediction) is the latest from the KTH group. It is an anatomically detailed model with conforming meshes of sulci and gyri with embedded white matter fiber tracts. The ADAPT model is equipped with a hierarchical image registration-based personalization pipeline that allows fast generation of detailed subject-specific models for almost any brains that have significant anatomical differences comparing with the baseline.^104^ The performance of the head model is evaluated by comparing model predictions with experimental data of brain–skull relative motion, brain strain, and intracranial pressure. The ADAPT model has been used in a subject-specific multiscale analysis of concussion.^124^

The University College Dublin brain trauma model (UCDBTM)^79^ has been used to study falls and sporting injuries, to evaluate helmet performance and to establish threshold metrics of concussion.^35,89,128^ It was shown that the model can predict the location of contusions in two reconstructed falls.^35^ Recent work has used this model to explore relationship between strain in white matter regions of interest and DTI abnormalities in 8 reconstructed TBI cases.^127^ A significant relationship was found between radial and mean diffusivity in fornix and shear strain in this region. The model was updated in 2020^149^ with an important feature being a low coefficient of friction between scalp and skull (0.06), which is based on dynamic experiments on PMHS heads.^148,149^

The SIMon (simulated injury monitor) head model was developed by researchers at NHTSA.^143^ Loading data from experiments on animals (114 experiments in total) were used to determine thresholds for strain, pressure and relative brain/skull displacement, which were proposed as predictors of DAI, contusion and SDH respectively. The SIMon Model was updated^144^ with improved anatomy and more elements to better preserve anatomical details (~ 46 k vs ~ 8 k elements). This model was calibrated for predicting the risk of DAI only. SIMon was used in later studies to develop the BrIC and UBrIC brain injury criteria.^46,142^

The Total HUman Model for Safety (THUMS) head model was developed by researchers at Toyota Central Research and Development labs.^95^ This model included various tissues, including scalp, skull, CSF, brain and meninges. In a recent version of the model, the brain, CSF and skull were connected with shared nodes, in contrast to a tied contact in the older version, to address computational instability issues^81^ and later an anisotropic material model was incorporated for the brain.^10^ This model was used to develop brain injury criteria based on rotational kinematics of the head (RIC and PRHIC).^94^ The THUMS is available in several anthropometries.

The Global Human Body Models Consortium (GHBMC) head model is derived in part from the GHBMC 50th percentile male model developed by an international consortium.^114^ It has been used to predict brain tissue response in automotive and sporting impacts.^40,46,103,139,142^ The GHBMC model has been used to develop brain injury criteria, BrIC and UBrIC, and a second-order system for predicting brain strain.^46,47,142^ It was later used to determine correlations between fifteen kinematics-based head injury criteria and strain in brain tissue in occupant and pedestrian crash tests.^45^ This study showed strong correlation between rotational kinematics and brain tissue strain. As with THUMS, several other sizes exist, including but not limited to the 5th percentile female and the 95th percentile male. The GHBMC model pedestrian model is approved for use with Euro NCAP *in silico* test guidelines for pedestrian protection which involve the calculation of TBI risk.

The Worcester Head Injury Model (WHIM) has been used to study concussion in sports.^12,86,115^ An early version was used to study concussed ice hockey and football players who had pre and post-trauma diffusion imaging scans.^115^ The model was used to predict strain and strain rate in the corpus callosum of the players, using acceleration data from instrumented helmets. They showed that both strain and strain rate in the corpus callosum correlated with changes in diffusion tensor imaging parameters of the corpus callosum. The subsequent isotropic WHIM V1.0 was used to study the reconstructed NFL concussion dataset using machine learning.^21,160^ White matter anisotropy based on whole-brain tractography from the same subject used to develop the model was later incorporated through the Holzapfel Gasser-Ogden constitutive model (anisotropic WHIM V1.0).^171^ This model also serves as the basis for deep learning models for (near) real-time regional or whole-brain responses.^55,158,159,161^ The model was recently further upgraded (anisotropic WHIM V2.1) to reach strain mesh convergence^170^ and to include the cerebral vasculature network and brain heterogenous material properties from magnetic resonance elastography.^173,174^ This model offers insight into strains of the cerebral vascular network in typical concussive impacts in sports and severe impacts in car crashes.

The Atlas Brain Model (ABM) was developed at Wake Forest University.^122,123^ This model includes cerebrum, cerebellum, and brain stem with special attention paid to similarity and interoperability with imaging atlases. The boundary conditions, including falx cerebri and tentorium cerebelli, are modeled in a matter to allow sliding. The CSF layer is tied to the brain and the boundary between the CSF and skull is defined to allow relative motion. The brain interacts with normal forces and against the skull and the falx and tentorium affecting its motion. The model includes gyri and sulci and was developed from an atlas of the human brain in Montreal Neurological Imaging (MNI) space by converting MRI voxels into hexahedral elements. This model was used to create and explore computational metrics of injury^118^ and has been used to explore pre to post season imaging changes in football players.^91,92,120,129^ Recently, this model was used to explore and report a large variety of whole-brain and more targeted injury metrics focused on tensile, shear and compressive strains,^118^ where strain-based injury metrics outperformed kinematic metrics in discriminating neuroimaging changes over the course of a season of football.^119^

The Imperial College (IC) FE model of the human head was developed using an image-based meshing method to incorporate fine details of the brain anatomy, including the sulci. By using this model, it has been shown that maximal strain and strain rate are concentrated in the depth of sulci in sporting collisions.^54,186^ The sulcal depths are the locations where pathology of chronic traumatic encephalopathy (CTE), has been found in athletes subjected to repetitive head kinematics. An analysis of the diffusion imaging of a large cohort of TBI patients also showed that white matter abnormalities are concentrated in sulci, providing converging evidence for the predictions of the model. This model has been used to evaluate new helmet technologies that are designed to manage the rotational motion of the head.^2,93,140^ Recently the model was upgraded to incorporate fine details of the venous system mapped from 7T QSM (quantitative susceptibility mapping) image of the same subject.^37^ This model was shown to be able to predict the location of small deposits of venous blood, known as microbleeds and a marker for axonal injury in mild TBI, seen in the SWI (susceptibility weighted imaging) scan of a rugby player following a head collision.

One model developed at Penn State focused more on progressing the computational methods used to bridge between FE modeling and imaging by developing a method to explicitly represent axonal fiber tractography,^49,50^ a technique now adopted by others. It has been used to study impacts in football as well as military overpressure loading.^51^

Two models were developed at the University of Virginia. The UVA-Axon model^155^ is based on the GHBMC M50 model with updated isotropic Holzapfel Gasser-Ogden brain material properties and with explicitly embedded axon tractography.^49^ This embedded axon technique was later incorporated into primate brain models to study interspecies scaling^156^ and a pig brain model to study the relationship between brain strain and axon pathology.^69^ They identified that global maximum axonal strain measured in the embedded axons was the best predictor of injury for all species and TBI severities studied, with global MPS-95 as a suitable alternative metric.^157^ Further, the UVA-Axon model has been used to study the functional changes that occur from TBI by evaluating the loss of functional network efficiency from localized regions of high strain.^8^

The second CAB-20MSym model^62^ was developed based on a template brain image from 20 young, healthy male participants.^130^ It was developed to integrate into the Registration-Based Morphing (RBM) technique to automatically generate subject-specific FE brain models through registration to preserve both external and internal neuroanatomical characteristics.^61^ A unique aspect of the model was that brain material properties were optimized to ensure the model is biofidelic across a wide-range of kinematic conditions using human volunteer magnetic elastography,^74^ volunteer tagged-MRI data,^101^ and human cadaver sonomicrometry data.^4^ The optimized model was then validated against additional sonomicrometry data and the final tissue properties were validated against independent tissue experimental data.

Most of these models represent a generic subject, typically that of a 50th percentile male head. It is known that a larger brain will produce higher strains than a smaller brain when applying the same head impact kinematics as model input.^62,100,105,109,156^ Therefore, caution must be used when applying generic models to a specific subject, especially for females and youth subjects, where greater differences in brain morphology relative to the generic model are expected. Conversely, additional work is necessary to develop advanced modeling and/or simulation strategies that will compensate for brain response differences due to morphological variations to improve subject specificity.^105,109^ A recent study identified that the size of the head and brain, tragion-to-top distance, as well as head length and width are the anatomic components that most significantly influence head geometric variations.^109^

## Model Validation

Validation is a process where the response of the model is compared to independently measured experimental data under the same applied loading conditions. It is critical to validate models against experimental data to establish the necessary confidence in prediction accuracy of the response variable of interest.^7^ Early brain models were validated against intracranial pressure data collected from human cadaveric impacts.^125,147^ However, brain pressure is not thought to be a mechanism relevant to brain tissue shear responses due to the assumed near incompressibility of the brain.^99,116^ In fact, brain pressure is directly related to the mass (volume/density) and shape of the brain when subjecting the head to linear acceleration.^19,176^ Therefore, a model only “validated” against pressure is not sufficient for predicting large brain deformation generated from shearing of brain tissue.

To probe the brain’s shearing deformation, relative brain-skull displacements were measured in human cadaveric heads. An earlier technique used high-speed X-ray that employed neutral density targets (NDTs) made from radio-opaque materials. They are sparsely implanted in the brains of human cadaveric head-neck specimens.^39,72,73^ The specimens are subjected to impact, and 2D in-plane marker motion is tracked. The use of a second X-ray system orthogonal to the primary X-ray source enables the visualization of out-of-plane marker motion, permitting measurement of 3D brain motion.^73^ The X-ray method requires a line-of-sight between the emitter, specimen, and camera to obtain a clear image, which can potentially limit the mounting hardware and the direction of loading for the tested specimen.

Sonomicrometry has also been used to measure dynamic 3D displacements of brain^3,4^ via trilateration and Kalman filtering.^5^ Because displacement is measured sonically rather than visually, this technique does not limit the external mounting hardware or the direction of specimen loading. Nevertheless, the measurement sampling rate is limited by the number of transmitting crystals. Finally, dynamic ultrasound has also been used to study peripheral brain motion.^113^ Limitations with this technique are the penetration depth of ultrasound waves, 2D tracking of motion, and the substantial disruption of the brain-skull boundary condition due to the craniectomy.

To validate a brain model, head kinematics are first prescribed for simulation. A common practice to assess model biofidelity is to compare model predicted relative brain-skull displacement trajectories with those from experiments. The degree of model-experiment agreement is then evaluated independently for each displacement component before an average score is calculated. If model-predicted displacements closely match with those from the experiment, then the model can be thought to be well validated against the available data. These assertions are subject to the limitations associated with experimental error, differences between human cadavers and live subjects, differences in anatomy between the experimental subjects and the models, and the biomechanical variation naturally occurring in the population.^172^ Complete agreement with all experimental data is unlikely in practice.

There are also efforts using marker-based displacements to derive brain strains for validation.^172,184,185^ Essentially, the strategy is to assess the aggregated consistency between model and experiment in terms of marker displacement components among multiple markers as a whole. The magnitude of these “averaged strains” are shown to be consistent with model predicted whole or averaged brain strains, suggesting that they are capable of discriminating model strain responses.^172,185^ Nevertheless, there is potential for displacement measurement error to propagate into sparse marker-based strains.^177^ In addition, the averaged strains may not have the spatial resolution to inform strain accuracy at the element level because the number of markers is limited, and they cannot be too close to each other in practice. Hypothetically, nonetheless, the averaged strains would converge to more localized counterparts when more embedded markers are available.

The inherent limitations of embedded sparse markers may preclude validation for strain prediction at the element level, regardless of the approach.^177^ Recent strain measurements from tagged MRI with human volunteers may mitigate some of the limitations, as they provide voxel-wise strains.^11^ However, their “effective” spatial resolution is similarly limited by spacing between tag lines and imaging planes.^22,101^ In addition, they are limited to impact severities far below injury, and extrapolation of data to injurious levels may be flawed given the brain non-linear responses. Nevertheless, peak rotational kinematics of these measurements (244–370 rad/s^2^) are on the same order relative to the 25th percentile sub-concussive rotational acceleration peak magnitudes (531–682 rad/s^2^).^132,168^ Therefore, these data have been increasingly used to validate brain models at low impact magnitudes,^48,86,154,172^ which may offer insight into brain biomechanics relevant to subconcussive kinematics.

The cadaveric and *in vivo* brain biomechanics data are all valuable in assessing the response of a brain model. However, a “gold-standard” strain field to validate a model for injury-level deformation prediction with typical FE element-level resolution in live humans does not exist.^11^ Objective rating methods such as CORA^52^ are commonly used during validation, but the specific use of these types of rating methods are not consistent across the field. In addition, the quality assessment may not be effective, as different versions of the same model can produce significantly different strains even if they have similar CORA scores.^155,172^

Given these considerations, we recommend a comprehensive validation approach against representative high- and mid-rate cadaveric experiments with varying impact direction, kinematic magnitude, and duration. For brain models intended to simulate low severity kinematics common in contact sports, additional validations against *in vivo* brain strains are also recommended,^168^ especially given the potential relevance of subconcussive kinematics to the onset of concussion.^133,141^ A comprehensive validation across a wide range of loading conditions is expected to maximize the confidence in deriving tissue-based injury metrics.^118^ For all validations, it is important that the model be scaled to match with the test subject’s head dimension, and ideally, the brain dimensions if such information is available.^6,172^

Finally, it is important to recognize that as new experimental data^4,39,65^ and analytic strategies^172,185^ become available, the validation quality of brain models should be reevaluated from time to time to maintain or improve the level of confidence in kinematic simulation.

## Model Simulation

After model development and validation, a brain model is ready for kinematic simulation. Typically, this is completed using a commercial FE solver,^1,110^ although open-source alternatives such as FEBio^111^ also exist. Most commercial codes can use explicit time integration schemes with parallel processing.

There are a host of numerical challenges that brain models face primarily due to the rapid loading to soft biological tissues. As previously described in Sect. 3, a typical material model for brain tissue is nearly incompressible, which leads to a Poisson’s ratio close to 0.5 (e.g., > 0.49999). As a consequence, this requires special numerical techniques to alleviate the numerical issues related to volume and shear locking.^29,63^ In addition, for typical brain models utilizing under-integrated hexahedral meshes, the elements can exhibit hourglassing, which are non-physical, spurious mesh deformation modes.^13,98^ Modern simulation packages allow brain modeling to be numerically accurate and stable. Nevertheless, modeling such a compliant, nonlinear and viscous material remains a challenge.

At a minimum, FE-based brain model systems should account for dynamics, the ability to model nonlinear viscoelastic materials (e.g., visco-hyperelastic), reduced integration with hourglass control or other elements free of locking. Furthermore, parallel computing capabilities, hexahedral based meshes and coordinate system transformations (i.e., body fixed vs ground fixed) are encouraged. Note that while wearable kinematic sensors may have the ability to measure repeated sub-concussive impacts with accuracy, there are currently no widely accepted methods for quantifying the history-dependent tissue damage that results from repeated loading.^41,53^

Applying boundary conditions is another numerical aspect needing attention. Conceptually, an impact transfers force to the head via contact with the helmet or ground. However, most sensor systems report linear and rotational accelerations instead of force or pressure. For impacts relevant to contact sports, the skull is usually assumed to be rigid because skull deformation is expected to be negligible. Therefore, skull motion is fully described by the linear acceleration and angular acceleration/velocity, such that impact location and direction become irrelevant for modeling purposes. This allows the kinematics to be commonly prescribed in terms of 6 degrees of freedom (DOF) of head acceleration (three for linear and three for rotational), or equivalently velocity, to the rigid body skull at the head center of gravity (CG). The head CG is the location where accelerometers are mounted in crash test dummies, and is often the location where wearable sensors measurements are transformed.

With the assumption of near incompressibility of the brain, linear acceleration induces little strain.^14,87,98^ As a consequence, 3DOF head rotational kinematics provide the majority of the information necessary to drive models for strain estimation. Linear acceleration does influence brain strain in the critical brainstem region when there is a large component in the inferior-superior direction,^96,97,161^ which should not be ignored in real-world applications. Even if pure linear acceleration produces limited strain, it can lead to injury. Therefore, mechanical variables sensitive to linear acceleration such as stress may also be considered as an alternative.

Kinematic information from wearable sensors should also be handled with care because they may not use a standardized orientation or naming methodology. For example, some sensor systems export data that have been transformed from the physical accelerometer and gyroscope readings embedded in a mouthguard situated on the teeth to the estimated head CG of the athlete. However, other sensor systems export data directly from the sensors without transformations. The burden then lies on the modeler to transform the data properly before kinematic simulation. Our group recommends sensor manufacturers to standardize their data export protocol. One reference for this may be the J211 coordinate system adopted in the automotive and military area.^131^ Another alternative is to follow that used in the medical imaging community, where the *y* and *z* positive directions are reversed relative to those in J211. In either case, it is necessary to define and use anatomical axes.

Wearable sensors measure the motion of the skull while the brain motion lags behind the skull. This phenomenon and the time-dependent deviatoric (i.e., time lag) material behavior of the brain tissue causes the brain to continue to deform in response to head impact in some cases even after significant skull motion ends.^4,83^ In some cases, this ‘post-impact’ brain tissue deformation can be larger than that experienced during the impact duration when the main skull motion occurs and the sensor data is collected. This post-impact brain deformation is also shown to be dependent on the brain geometry and is more significant in larger brains.^107^ Current head kinematic sensor systems collect data for approximately 50–200 ms which contain pre-trigger and post-trigger times and the trigger time point is typically considered when the resultant linear acceleration exceeds a threshold value. When dealing with data near the lower end of 50 ms, the simulation input can end, while the brain tissue may continue to deform. Therefore, we recommend simulations to encompass a longer time window than the impact duration and check for significant brain strains ‘post-impact’. A recent study recommends a 20 ms pre-trigger and 70 ms post-trigger duration for a proper capture of brain deformation during the full kinematics.^107,174^ From a simulation perspective, another study recommends at least ~ 20 ms additional simulation time (with zero acceleration) for the deep brain regions to reach peak strain.^83^ .

### Impact Simulation Efficiency

While great strides have been made over the past half century to improve the sophistication and biofidelity of head injury models,^112,162^ relatively less attention has been paid to improve simulation efficiency. Direct impact simulations using a validated brain model may be the most accurate, but substantial computational runtime may make them less feasible for real-time monitoring and assessment. This is important and especially relevant to contact sports, as each player typically sustains dozens to hundreds of impacts in a play season and there is growing concern on the cumulative effects from many subconcussive kinematics on the onset of concussion.

To achieve rapid estimation of brain response, several approaches have been pursued. One method is to develop generalized kinematic-based tissue deformation hypersurface through parametric simulations.^47,68^ This allows brain strain to be quickly elucidated from kinematics. However, the idealized acceleration pulses used for simulation may not capture details of realistic accelerations. Another limitation is that they typically estimate MPS of the whole brain; thus, losing the details of the spatial distribution. An alternative approach is a pre-computed brain response atlas based on simulations of idealized rotational pulses using discretized parameters of head impact (i.e., peak magnitude and duration, rotational axis azimuth and elevation angles). Although this method allows element-wise brain strains to be efficiently interpolated or extrapolated,^84^ accuracy may suffer when kinematics are more complex.^175^

Recent deep learning-based approaches avoid these limitations, as they retain high accuracy without the need to simplify the brain model, response output, or kinematic input. These models use kinematic profiles directly^16,55,158^ or extracted features^164^ as input, and employ thousands of direct model simulations based on realistic kinematic profiles to generate training data. Upon convergence, the trained deep learning models can instantly estimate detailed strains^55,164^ or maximum stress of the brain and injury risk directly.^16^ Recently, a transformer and a separate convolutional neural network have been developed to rapidly estimate the complete spatiotemporal details of brain strains with high accuracy (normalized RMSE 2–3% with *R*^2^ > 0.99).^159^

Given these results, deep learning models may have the potential to allow monitoring detailed brain strains from many head impacts and for a large number of athletes in diverse contact sports teams. With high accuracy relative to direct model simulation, deep learning models could also promote model sharing by effectively shielding the details of FE model construction from end users. This may be especially helpful when it is not practical to share the biomechanical model, including the mesh elements and nodes, itself. An added benefit is that they do not need any specialized FE code or sophisticated computing hardware, and computation time is drastically reduced. Nevertheless, a large number of training samples are necessary to yield a desired accuracy, which would demand a enormous initial cost of simulation. This could be mitigated by transfer learning (i.e., reusing network weights instead of random initialization) to reduce the samples required, even when the technique is applied to a different brain model or impact type (e.g., automotive vs. contact sports).^159,161^

### Brain Response Sensitivity to Impact Kinematics

Errors in kinematics are typically assessed by the percentage of peak magnitudes of linear and rotational acceleration and rotational velocity.^108^ A potential limitation is that this approach is unable to assess the significance of other important kinematic features on the resulting brain responses, such as direction of rotation,^4,96,97,152^ area of the brain that experiences high strains or stresses,^98,115^ temporal variation,^14,169^ variation by direction or orientation of brain tissue,^26,57,59,86,154^ and even digital filters that are known to affect strain magnitude and distribution.^90^ We are in the adolescent stage of exploring these variations, with many promising findings that are helping researchers better understand the biomechanics they are encountering in the field. Determining how kinematic errors affect brain response may seem straightforward, e.g., by comparing the simulated brain responses to reference data using a validated brain model. Nevertheless, this process can be made much more efficient when using a deep learning model.^108^

## Model Simulation Results and Interpretation

There are several considerations related to model result interpretation in the context of head impact simulation using kinematic data measured from head acceleration sensors. Assuming sufficient confidence has been achieved regarding the model development and biofidelity, there are many metrics that are commonly used by the brain modeling community for quantifying head kinematic severity. The aphorism “All models are wrong, but some are useful” is often attributed to the statistician George Box,^18^ and surely applies to FE modeling of the brain, one of the most complex human anatomical structures.

A main objective for the creation and use of brain models is to better interpret head kinematics in the context of injury risk. This involves calculation of FE based metrics for subsequent quantification of impact exposure, risk estimation, or other activities. Frequently, these results are used to relate to contextual and outcomes information. Contextual information here is defined as information which may be useful to coaches, athletes, parents, regulatory bodies, equipment designers, *etc*. Outcome information generally refers to clinically and scientifically useful information relating to maximizing benefit and reducing risk. This can be neurocognitive,^154^ imaging,^28,115,129,163^ or biomarker data,^64^ *etc*. There is long-term potential for FE brain model results to improve our understanding of pre- and post-exposure changes of various clinical biomarkers, and better utilize head kinematics to predict clinically relevant outcomes typically interpreted by the engineer, physician, athletic trainer, public health scientist, *etc*.

In a sense the FE modeling community is at the early stages of understanding and utilizing the tissue-level metrics that can be computed using brain models. They represent an example of the pareto principle—the majority of the value can be achieved with less effort, and the remaining value will require pushing the boundaries of thought and expertise, is the purview of researchers, and holds the promise for better capturing nuance and serving as a better context or outcome discriminator. These activities are expected to lead to a better fundamental understanding of the biomechanics of kinematic exposure and acute or chronic injury. Considering that there is known asymmetry in the brain both anatomically and functionally,^36^ the brain changes structurally with age,^33^ and is a networked system that is structurally and functionally complex,^20^ no FE modeling approach totally represents the anatomy or function of the brain completely. Further, no FE model allows tissue-level interpretation of even the most basic functional, biochemical, or molecular outcomes observed in neurotrauma models. Most biomechanics researchers studying the brain focus on trying to approximate the mechanical response of the parenchyma caused from head impact, using the best available neuroanatomy, material property, and boundary condition data. The outcomes of these simulations are complex, but metrics are used to simplify the simulated outcomes to allow quality analyses to be performed.

There are basic metrics commonly adopted in brain models, beginning with simple strain measurements.^121^ Peak maximum principal strain and stress are some of the earliest explored metrics from a test and FE modeling standpoint.^73,98^ The history and experience with modern, FE-based approaches has rapidly expanded over the past couple of decades, allowing for a slew of other metrics.

Scalar values may come from one element of the brain at one specific time in the simulation, often the time of peak maximum principal strain and strain rate or their product, at any point during the simulation.^27,87,115,172^ Most FE models provide this scalar value. Nevertheless, the peak or highest strain in an FE brain may be spurious. Therefore, this measure may not discriminate impacts well. To mitigate the issue, it is possible to adopt use of the 5th percentile compressive strain or the 95th percentile tensile strain.^43,118^

Volumetric based metrics are also scalars, which have the advantage of describing the state of the entire brain. As with percentile rank based metrics, these are best thought of as deriving from the complete picture of the stress or strain state at a moment in time or at any time during the kinematics, with functional similarity to CSDM but without the threshold. These are a product of the volume of each element and the peak strain experienced by that element, representing the entire volume of brain tissue.^118^

Percentile based metrics and volumetric metrics have been derived and can be developed for strains (typically first principal strain or maximum tensile, third principal strain or maximum compressive, and shear strain) and strain rates, which are the maximum rate of change of each of the above.^118^ Metrics like the CSDM^144^ have been derived by choosing thresholds thought to be biomechanically or clinically relevant from these basic metrics.^66,70,86,106,167,177^ Threshold based metrics have the benefit of representing or are associated with relevant and helpful values of risk, but also have the drawback that they sometimes combine aspects of continuous and categorical values, measuring zero below a certain threshold and varying above the threshold. In this sense they may be more practical but may be less helpful for research designed to identify thresholds from clinical data when one is not known.

These scalar values may be oversimplified as they lose information on the anatomical location of where peak strains occur. In these cases, the brain models may be underutilized. At the other extreme, elementwise strains can also be used; but this may be excessive as neighboring elements typically have similar strain magnitudes. As a compromise, therefore, it may be advantageous to report peak strains in a selected few anatomical regions, for example, the left and right cerebrum, corpus callosum, cerebellum and brainstem.^161^ The resulting response “vector” is analogous to the Injury Severity Score used to encode AIS of the six body parts. This idea can be further extended by considering peak strains in gray matter regions and their connections, in which case a response “matrix” can also be obtained.^160^ In short, model result interpretation is important for the best use of brain model simulations and efforts are converging towards more sophisticated metrics than a scalar metric previously used.

### Model-Based Injury Prediction

The utility of a validated brain model ultimately rests on how it performs when predicting the likelihood and extent of injury in the real world, including identifying the location of the injury within the brain. Therefore, verifying injury predictions based on model simulated responses against clinical injury manifestations such as neuroimaging and symptoms is helpful.^37,54,77,115^ However, challenges remain, due to inherent confounding factors associated with human subject injury (e.g., concussion history and accumulation of subconcussive impacts), uncertainties in modeling parameters (e.g., material properties and brain external and internal boundary conditions), and limited real-world cases that provide both accurate impact kinematics and injury findings on the same subjects. Consequently, these efforts remain at an early stage. Nevertheless, increasing the availability of real-world injury cases and public accessibility is important to the modeling community to test the utility of the diverse brain models.

An ultimate use of a brain model is to estimate risk of injury, predict injury, or to quantify subconcussive kinematic exposure as previously discussed. It has been anticipated that FE model simulated regional strains metrics may improve the performance in injury prediction over simple kinematic injury metrics Another application of model responses is to derive injury risk functions, which are anticipated to be more effective than those based solely on kinematics. In fact, peak MPS of the whole brain currently serves as the benchmark to assess the effectiveness of kinematics-based injury metrics. Conventionally, brain strains such as peak MPS of the whole brain and CSDM as well as stress have been used to fit a univariate logistic regression model using simulation results from reconstructions of real-world cases. An injury risk function is then developed that best separates the injury vs. non-injury cases, e.g., using SUFEHM,^42,105,173^ WSU,^171^ KTH,^138^ and GHBMC.^78,148^ However, a major concern with these injury risk functions is the “selection bias” due to the severe under-sampling of noninjury cases. This potentially would result in gross overprediction of the risk of injury for real-world impacts.

This limitation is partially alleviated by incorporating laboratory-based animal injury data and animal FE brain models that are developed using methods consistent with the human models. Recently, injury-risk functions using the GHBMC^114^ and UVA-Axon^155^ models have been developed using human, primate, and pig injury data, through the identification of species-independent tissue metrics MPS and maximum axonal strain.

Another concern with utilizing injury data is the uncertainty of the actual clinical diagnosis, or the lack of neuropathology or neuroimaging data that can help support the FE model predictions (again, partially alleviate when incorporating animal data).^34,44,67^ Furthermore, injury risk data do not consider the potential reduction of injury tolerance from cumulative head impacts or previous injury. Therefore, caution must be exercised when using these risk functions for the general assessment of concussion in a sport context.

More recent efforts have employed feature-based machine learning,^146^ where multiple response features in different anatomical regions are combined to predict the likelihood of injury.^21,160^ They have been shown to improve injury prediction performance relative to other scalar values, such as peak kinematics or peak MPS. In addition, testing performances from leave-one-out or *k*-fold cross-validation may be more objective in performance comparison than fitting performances commonly used in training logistic regression models. Otherwise, a 100% sensitivity and specificity fitting performance can be achieved,^21^ which is not meaningful in practice. From data science perspective, it is critical to use an objective testing performance rather than a fitting or training performance for assessment.

### Response Comparison Across Models

Response comparison across diverse range of brain models used to assess head impacts is a recurring issue, and two models may predict different levels of severity for the same head kinematics.^42,60,82^ Ideally, all models predict the same injury risk, but disparate results are possible because most models have been developed in isolation. Assessing the differences in scalar or vector strains is straightforward. However, assessing the difference for the whole brain may be non-trivial because different models may have different meshes, or spatial sampling of the deformation field. To manage mesh-mesh mismatch, one approach is to resample the simulated results into a standard image format, such as the MNI space.^85^ A standard image representation of the simulated responses across diverse brain models may allow seamless comparison of simulated responses, or to directly compare with neuroimage findings for a given subject. In addition, an image representation of the response field may promote a more convenient data sharing strategy in the future, as it effectively eliminates the need for explicit description of the brain model and mesh.

To facilitate a convergence of model responses and to promote the continual development of brain models, the authors recommend an open-source digital repository to archive impact simulations of well accepted validation experiments and reconstructed real-world cases from existing brain models. In addition, idealized or simplified head kinematics can also be used for benchmarking purposes to compare simulated brain responses across different models. The repository may serve as a first step towards harmonizing various brain models to promote the development of new or upgraded models in the future.

## Discussion/Conclusions

One of the main focuses of the TBI research field is related to the biomechanics of the brain from head kinematic exposure in contact sports, and the subsequent risk of injury. Early biomechanical studies relied on interpreting head kinematics, such as linear and rotational acceleration peak magnitudes, to quantify impact severity and to assess the likelihood of injury. With the advent of sufficient computing power accompanied by the possibility of simulating physical events, FE models of head and brain have emerged,^162^ first of simplified anatomies with two dimensional and coarse meshes. They have continually improved and advanced over the past half century^112^ with increasing sophistication in brain anatomical representations, material properties reflecting complex mechanical responses of the brain parenchyma, and advanced modeling capabilities to estimate tissue mechanical behaviors in specific anatomical regions such as corpus callosum and sulci, and along dense white matter fiber tracts and cerebral vascular network. These models have suggested tissue-level injury thresholds and enable translating head kinematics into detailed biomechanical responses of the intracranial tissue thought to be responsible for injury.

This article reviews brain model development, validation, impact simulation and result interpretation primarily in the context of contact sport. Because brain models can predict physical tissue responses, there is general consensus within the modeling community of their strong potential to study the biomechanics of head impact exposure. Best practices in model development such as meshing and simulation numerical considerations will continue to progress. FE brain models will continue to advance and to reflect updated understanding of brain mechanics when relevant experimental data become available. Nevertheless, there are also an array of issues and challenges beyond model development that are relevant for impact monitoring in contact sports.

On the high level, the first challenge might be a robust model validation to ensure sufficient biofidelity for a large range of impact conditions that occur in contact sports. This is also related to model development for assignment of brain material properties and brain-skull boundary conditions, where validation is necessary to confirm their effectiveness. While most head kinematics experienced in sports are of low magnitude and subconcussive, more severe impacts can happen that could lead to acute injury. Experimental data already exist from *in vivo* volunteers to mid- and high-rate cadaveric impacts to allow validating a model across the large kinematic severity spectrum. Nevertheless, efforts to utilize all of the available data for comprehensive model validation remain limited at present.

The second challenge is model simulation efficiency of head kinematics. Contemporary models often take hours per simulation on a high-end computing platform, and thus, cannot provide near-instantaneous results. This may be especially relevant for impact exposure monitoring in contact sports given that each player usually sustains dozens to hundreds of impacts throughout a play season and that each impact seems to be relevant given their potential cumulative effects. Advanced deep learning models have shown promise to efficiently estimate tissue responses^16,55,158,164^ and with high accuracy^159^ on a low-end computing platform. This may be valuable for continual exploration of cumulative injury metrics using tissue-level responses and to examine how they correlate with long-term clinical neurological findings.^119^ Nevertheless, similar to FE brain models, accounting for head/brain morphological differences in these deep learning alternatives is warranted.

In addition, the accuracy of simulated or estimated brain responses would necessarily depend on the quality of kinematics that serve as input. Therefore, concerted efforts from the head acceleration sensor and modeling communities are important to maximize the effectiveness in impact exposure monitoring. Standardized protocols and data formats to characterize head kinematics across sensor manufacturers as well as simulated brain responses from diverse models would facilitate these activities. Translating variations in head kinematics into variations in brain responses may provide more relevant context for result interpretation and evaluation than simple variance in peak kinematics.

Finally, a major roadblock for effective real-world use of brain models is the lack of sufficient kinematic and injury data encompassing sports, sex, age and other factors. This type of database is necessary to establish a relevant tissue response-based metric for injury detection and interpretation, as well as to quantify subconcussive exposure due to the potential cumulative effects on the onset of concussion.

Therefore, it seems clear that it is important to continue the collection of high-quality head kinematic data along with associated information on subject-specific age, sex, head morphological measures and clinical indicators of injury. Together with an efficient modeling scheme, eventually, these efforts may allow training brain models for more effective mTBI detection and monitoring of head kinematic exposure for potential clinical applications. By hosting the kinematic data and the resulting brain responses in a dedicated, transparent, and open-access data repository whenever possible, an improved understanding of the biomechanics behind concussion and subconcussive exposure may ensue. These efforts could contribute to enhanced mTBI mitigation strategies and rule changes to better protect the brain.

Based on the reviews and discussions, our group has compiled recommendations and consensus statements presented at the beginning of the paper.

## Abbreviations

ADAPT: A detailed and personalizable head model with axons for injury prediction
BrIC: Brain injury criterion
CAB: Center for Applied Biomechanics
CG: Center of gravity
CORA: Correlation Analysis Score
CSDM: Cumulative strain damage measure
CSF: Cerebrospinal fluid
DAI: Diffuse axonal injury
HIC: Head Injury Criterion
IC: Imperial College
ISO: International Standards Organization
FEBio: Finite element analysis software for biomechanics and biophysics
GHBMC: Global Human Body Models Consortium
KTH: Kungliga Tekniska Högskolan
NFL: National Football League
NCAP: New Car Assessment Programme
NHTSA: National Highway Traffic Safety Administration
MAS: Maximum axonal strain
MNI: Montreal neurological imaging
MPS: Maximum principal strain
PMHS: Post Mortem Human Subjects
RWE_CP_: Risk-weighted cumulative exposure
SDH: Subdural hematoma
SIMon: Simulated injury monitor
SUFEHM: Strasbourg University FE Head Model
TBI: Traumatic brain injury
THUMS: Total Human Model for Safety
UCDBTM: University College Dublin Brain Trauma Model
WHIM: Worcester Head Injury Model
WSUHM: Wayne State University Head Model
